# Associations between Water Supply Interruptions and Water Use, Drinking Water Quality, Child Health, and Caregiver Stress in Peri-Urban Malawi

**DOI:** 10.1021/acs.est.5c08820

**Published:** 2026-01-15

**Authors:** Caitlin G. Niven, Benjamin Clark, Emily Floess, Blessings Chirwa, Monica Matekenya, Emma Budden, Stella Cadono, John Chavula, Victor Chisamanga, Aubrey Dzinkambani, Chisomo Kaponda, Neema Ngondo, Norah Patterson, Sheena Symon, Brighton A. Chunga, Rochelle H. Holm, Petros Chigwechokha, Francis L. de los Reyes, Cassandra L. Workman, Angela R. Harris, Ayse Ercumen

**Affiliations:** † Department of Forestry and Environmental Resources, 6798 North Carolina State University, Raleigh, North Carolina 27695, United States; ‡ Department of Civil, Construction, and Environmental Engineering, North Carolina State University, Raleigh, North Carolina 27695, United States; § Department of Water and Sanitation, 107930 Mzuzu University, P/Bag 201, Luwinga, Mzuzu 105203, Malawi; ∥ Northern Region Water Board, Bloemwater Street, Kawiluwilu House, Private Bag 94, Mzuzu 105200, Malawi; ⊥ Department of Plant and Microbial Biology, North Carolina State University, Raleigh, North Carolina 27695, United States; # Department of Biological Sciences, 542087 Malawi University of Science and Technology, P.O. Box 5196, Limbe 312229, Malawi; ∇ Center for Healthy Air Water and Soil, Christina Lee Brown Envirome Institute, School of Medicine, 5170 University of Louisville, 302 E. Muhammad Ali Blvd., Louisville, Kentucky 40202, United States; ○ Department of Anthropology, University of North Carolina Greensboro, Greensboro, North Carolina 27412, United States

**Keywords:** water interruption, water intermittency, intermittent water supply, water insecurity, hygiene, water quality, E. coli, antimicrobial resistance, diarrhea, respiratory infection, stress

## Abstract

Interrupted water supplies contribute to water insecurity, water quality risks, diarrhea, and stress; other risks (acute respiratory infections (ARIs), antibiotic use, antimicrobial resistance) remain unquantified. We assessed associations between water interruptions and various outcomes. Among 237 households in Malawi, we conducted a cross-sectional questionnaire and tested drinking water for generic and cefotaxime-resistant *Escherichia coli* (CREC). Water interruptions in the past month were reported by 32.5% of households; interruptions were unpredictable and more common in piped supplies. Households with interruptions were 3−5 times more likely to be water-insecure, skip laundry and handwashing after handling animal feces (*p*-values < 0.05). Most water samples came from storage, and 65.7% harbored *E. coli* and 8.4% harbored CREC; households with vs without interruptions had similar fecal contamination. Children <5 years experiencing interruptions had increased caregiver-defined diarrhea (prevalence ratio [PR] = 1.85, 1.02−3.37) and ARI with fever (PR = 1.98, 1.09−3.57). Rare (1−2/month) interruptions were associated with diarrhea and antibiotic use; frequent (≥3/month) or long (above-median duration) interruptions with ARI. Frequent or short interruptions were associated with stress. Our findings highlight respiratory risks from water interruptions and suggest that interruption frequency and duration may influence enteric vs respiratory pathogen transmission through distinct mechanisms. Prospective studies should validate these associations and evaluate mitigation strategies.

## Graphical abstract

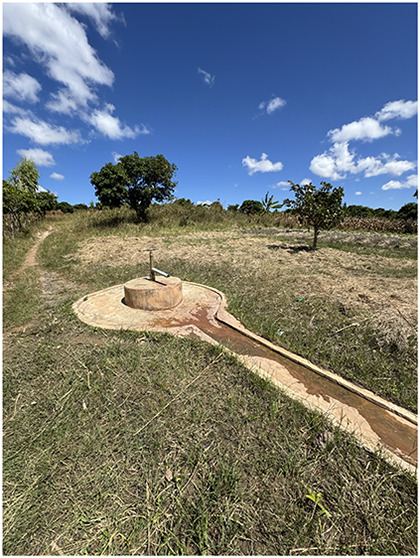

## Introduction

The United Nations Sustainable Development Goals aim to achieve universal access to safe and affordable drinking water; meeting this goal remains a global challenge.^1^ The WHO/UNICEF Joint Monitoring Programme (JMP) defines a safely managed water service as water from an improved source that is on-premise, free of fecal and priority chemical contamination, and available when needed.^1^ They define “available when needed” as households reporting their water is “sufficient” or available “most of the time” (at least 12 h/day or 4 days/week).^2^ The WHO emphasizes that an improved water source must be continuously available on premises.^3^ However, intermittently operated piped supplies, where water is delivered at intervals,^4^ serve >1 billion individuals, 12.5% of the global population.^5^ Piped supplies also experience transient interruptions due to pipe or pump breaks, power outages or acute water shortages,^6^ while nonpiped supplies such as boreholes can have prolonged interruptions due to handpump malfunction, water scarcity or both.^7^

Interrupted water access contributes to water insecurity (i.e., inability to access adequate, reliable, and safe water for everyday use, including drinking, washing, cooking, and cleaning),^8,9^ with implications for water quality, hygiene, health, and emotional well-being. Both chronic interruptions in intermittent systems and transient interruptions in continuous systems are associated with compromised water quality^10^ and enteric infection risks,^11^ while evidence has also emerged on stress outcomes,^12^ especially if interruptions are unpredictable.^13^ Other potential risks, such as respiratory infections from impaired hygiene during interruptions, increased antibiotic use to treat infections, and antimicrobial resistance, have not been studied. Therefore, current assessments may underestimate the global disease burden from water supply interruptions.

Supply interruptions affect drinking water quality through multiple pathways.^14^ For piped supplies, a lack of pressure during interruptions allows pathogen intrusion. Microbiological contamination levels can be up to 45 times higher in intermittently supplied taps, compared to continuously supplied taps.^15^ Intermittencies can also force households to store water for extended periods, leading to contamination during collection or storage, or supplement their primary supply with unsafe/unprotected sources, which may be more heavily contaminated.^16^ While the evidence of water quality effects of supply interruptions is mostly based on conventional fecal indicators, antimicrobial-resistant bacteria are increasingly detected in global drinking water supplies and may disproportionately affect interrupted supplies through intrusion and point-of-use contamination. Water supply interruptions may also increase selective pressure for resistant strains (e.g., via heavy metals corroding from stagnant pipes during interruptions and acting as coselectors).^17^ Studies have detected antimicrobial-resistant bacteria in water supplies characterized as intermittent but lacked a nonintermittent comparison group to isolate the causal role of intermittencies in drinking water contamination with antimicrobial-resistant bacteria.^18–20^

Microbially contaminated water in interrupted supplies can lead to waterborne enteric infections (e.g., diarrhea). Further, water intermittency and limited water availability can contribute to water-washed infections by impeding essential hygiene practices (e.g., handwashing), as households may prioritize the use of clean water for other tasks. Water-washed diseases (e.g., enteric, eye, and skin infections) are transmitted due to a lack of sufficient water to maintain hygiene, and a lack of handwashing can also increase the risk of acute respiratory infections (ARIs).^21–24^ Studies have investigated the effects of water intermittencies on COVID-19 prevention measures and infection rates,^25–27^ but broader evidence on respiratory infections is lacking. Further, diarrhea and ARIs are major drivers of antibiotic use.^28–30^ Therefore, increased enteric and respiratory infections associated with water interruptions can plausibly lead to increased antibiotic use and consequently increase the community carriage of antimicrobial resistance through selective pressure. Water interruptions may also increase the carriage of antimicrobial resistance through the ingestion of drinking water contaminated with antimicrobial-resistant bacteria. No studies to date have assessed associations between water supply interruptions and antibiotic use or antimicrobial resistance.

The effects of lacking continuous water access extend beyond physical health and can significantly impact mental and emotional health through increased psychosocial stress and emotional burdens. Daily challenges in finding an adequate water supply, ensuring sufficient water is stored, and reserving or rationing water can contribute to chronic stress and anxiety.^13,31–35^ Women and girls are primarily responsible for water-related chores, particularly in sub-Saharan Africa, and bear the greatest burden.^34,36,37^ Studies in sub-Saharan Africa have found women must balance responsibilities with the burden of water-related chores such as fetching, treating, storing.^38,39^ Chronic stress, anxiety, and a sense of helplessness are common among those facing uncertain access to safe and sufficient water.^32–34,37,40^ Moreover, water insecurity does not exist in isolation, it is compounded by other stressors such as lack of access to safe sanitation, increased risks of waterborne diseases, conflicts over scarce resources and food insecurity.^41,42^ The cumulative impact of these challenges, including heightened disease risks, physical demands, and emotional strain, demonstrates that studies of water insecurity, including interrupted water access, should account for both physical and mental health outcomes to fully quantify public health impacts.

Malawi is a landlocked country in Southern Africa with a population of over 20 million, ranking among the world’s poorest 8% of countries.^43^ More than half the population lives in poverty, and one-fifth in extreme poverty,^44^ with over a quarter unable to afford the recommended daily food intake.^45,46^ Water insecurity is also widespread: 3.8 million Malawians lack access to clean drinking water, with rural access (65%) lagging behind urban areas (90%).^45^ Previous studies in Malawi have highlighted several challenges in water services. Piped water systems face sustainability issues due to logistical, technical, and infrastructural constraints.^47^ In informal settlements in Lilongwe, the capital city, limited and unreliable water kiosks force households to endure long queues and service gaps.^48^ At the utility level, water boards struggle with high levels of unaccounted-for water, mainly from leaks, undermining supply reliability.^49,50^

We aimed to examine the relationship among water supply interruptions, coping behaviors (water use, hygiene), intermediate outcomes (water insecurity, drinking water contamination with fecal indicators and antimicrobial-resistant bacteria), and physical and mental health outcomes (diarrhea, ARI, antibiotic use, stress) within peri-urban Malawian households. By investigating multiple nodes along the causal chain, we aimed to demonstrate the full health burden associated with interruptions and explore the mediating mechanisms behind specific outcomes.

## Materials and Methods

We systematically enrolled 237 households in Bangwe, a peri-urban town in Blantyre, Malawi. We used GIS to select random starting points within town boundaries and systematically approached every third household in each direction to ensure representative geographic coverage. We aimed to enroll households in geographic groupings (clusters) corresponding to informal neighborhood delineations. Households were eligible for enrollment if they had at least one child <5 years, and an adult caregiver >18 years was available and consented to participate. Enrolled households were visited once to conduct a structured questionnaire and collect drinking water samples.

### Data Collection

Trained enumerators from Mzuzu University (MZUNI) and Malawi University of Science and Technology (MUST) administered a questionnaire among the primary caregivers in the enrolled households. Water interruptions were enumerated using one of the 12 items on the Household Water InSecurity Experiences (HWISE) scale. The HWISE scale assesses the frequency of experiences related to water insecurity and associated negative emotional responses, using a five-point Likert scale from never (0) to always (20+ times) in the last 4 weeks.^9^ One HWISE item specifically asks how often the household’s primary water source was interrupted or limited in the last 4 weeks. We used this question to assess the occurrence and frequency of interruptions. We asked an additional question on how long the last interruption lasted before service was restored to separately assess the duration of interruptions, and whether respondents were notified or able to anticipate the interruption to assess the predictability of interruptions. We recorded coping behaviors, such as drinking water storage and use of secondary sources. We assessed handwashing at key times with an unprompted question (i.e., asking households to list all occasions when they wash hands without providing answer options to prompt them).

We recorded caregiver-reported health outcomes for all children <5 years in enrolled households, including diarrhea (as defined by caregiver), loose stools, constant cough, difficulty breathing, and fever within the last 7 days, and antibiotic use in the last 4 weeks. Antibiotics are commonly used to treat pediatric diarrheal and respiratory infections in low-income countries;^51,52^ we did not record reasons for antibiotic use. We recorded ear infections and rashes as negative control outcomes that are theoretically not associated with water interruptions.^53,54^ We recorded respondent stress using the 10-item Perceived Stress Scale (PSS) that evaluates feelings and thoughts in the past month as they pertain to how unpredictable, uncontrollable, or overwhelming life events are using a five-point Likert scale from never (0) to very often (4).^55,56^

### Sample Collection and Processing

Enumerators trained in the aseptic technique collected drinking water samples from enrolled households. Enumerators asked the respondent for a glass of drinking water the same way they would serve a child. Approximately 200 mL of sample was transferred into a sterile Whirl-Pak bag that contained sodium thiosulfate to remove any residual chlorine (Whirl-Pak Filtration Group). Enumerators recorded whether the sample was obtained from a tap or storage container, the source of the water, and whether it had been treated to make it safer for consumption. Field blanks were collected by placing a WhirlPak bag prefilled with 100 mL of sterile DI water into the field cooler of a randomly selected enumerator and asking them to return it to the laboratory along with the samples. One field blank was collected for approximately every 30 water samples (3.0%, 7/236). Samples were transported on ice to the MUST laboratory and processed within 5 h.

We used IDEXX Quanti-Tray/2000 with Colilert-18 media (IDEXX Laboratories, Westbrook, ME) with and without antibiotic supplementation to enumerate the most probable number (MPN) of *Escherichia coli* and cefotaxime-resistant *E. coli*. Cefotaxime-resistant *E. coli* was used as a presumptive indicator for extended-spectrum β-lactamase (ESBL)-producing *E. coli*, which are of concern due to their resistance to broad-spectrum β-lactam antibiotics and form the basis of the WHO’s Tricycle global surveillance initiative.^57^ 100 mL of sample and Colilert media were mixed in a sterile WhirlPak, then dispensed into a Quanti-Tray/2000. A second 100 mL aliquot from the same sample was analyzed using the same process plus the addition of 80 μL of 5 mg/mL filter-sterilized cefotaxime solution.^58^ Lab blanks were analyzed using the same steps with 100 mL of sterile DI water. One lab blank was processed for approximately every 10 water samples (11.9%, 28/236). Following incubation for 18 h between 35 and 36 °C, we counted the wells that were yellow and fluoresced under long-wave ultraviolet (UV) light, indicating the presence of *E. coli*.

### Ethics

The research protocol was approved by the Research Ethics Committee at Mzuzu University (MZUNIREC/DOR/24/38) and the University of North Carolina, Greensboro (20–0245), with additional ethical oversight from North Carolina State University via Inter-Institutional Agreement. All participants provided written informed consent in their local language (Chichewa).

### Statistical Analysis

#### Exposure Variables

We considered the occurrence, frequency, and duration of water interruptions. Using the HWISE question, we generated a binary variable for whether the household experienced any water interruptions and a categorical variable for interruption frequency (none, rare, frequent). We defined “rare” as 1–2 times in the last 4 weeks as per the original HWISE category. We combined the HWISE categories of sometimes (3–10 times), often (11–20 times), and always (20+ times) to define “frequent” because the latter two categories had few observations. We used the reported duration of the last interruption the household experienced to generate a categorical variable for interruption duration (none, short, and long). We defined “short” as below-median and “long” as above-median duration; we chose the median as the cutoff to split the data into evenly sized strata for analysis. We assessed associations between outcomes and each exposure variable individually; we could not assess associations with cross-categories of exposures (e.g., long and frequent interruptions) due to the small sample size.

#### Outcome Variables

Outcomes across the causal chain included coping behaviors (hygiene, water use practices), intermediate outcomes (water insecurity, water quality), child health, and caregiver stress (Figure S1).

##### Water Insecurity, Hygiene, and Water use Practices

We summed the scores of individual HWISE questions to calculate the composite HWISE score, including the full score across all 12 indicators and a truncated score excluding the interruption question. We generated a binary variable to classify households as water-insecure (HWISE score ≥ 12 out of a maximum of 36).^59^ We used individual HWISE questions to generate binary variables for whether households skipped specific hygiene practices 3+ times in the last month. We generated binary variables for whether households reported handwashing at specific key moments, stored drinking water, or used secondary sources.

##### Water Quality

We tabulated the prevalence and log-10 transformed most probable number (MPN) of generic and cefotaxime-resistant *E. coli*. We replaced nondetects with half the lower detection limit (0.5 MPN/100 mL)^60^ and values above the upper detection limit (2419.6 MPN/100 mL) with 2420 MPN/100 mL. For samples that were positive for cefotaxime-resistant *E. coli*, we calculated the relative percent abundance as the ratio of cefotaxime-resistant *E. coli* counts to generic *E. coli* counts for the same sample.

##### Child Health

We tabulated the caregiver-reported 7-day prevalence of diarrhea, ARI, ARI with fever, and negative control outcomes (ear infections, rashes). Caregiver-defined diarrhea was based on the caregivers’ response to the question whether their child had diarrhea, which captures their perception of what constitutes illness in their child and may drive treatment choices (e.g., seeking antibiotics).^61^ WHO-defined diarrhea was classified as ≥3 loose or liquid stools within 24 h, based on caregiver-reported symptoms. ARI was defined as a constant cough coupled with panting, wheezing, or difficulty breathing; ARI with fever included the same symptoms with fever occurring in parallel.^21^ We generated a binary variable for whether the child took antibiotics in the last month and tabulated how many times the child took antibiotics in that period.

##### Caregiver Stress

We summed the scores of individual PSS questions to calculate the composite PSS score and generated a binary variable for high stress (PSS score ≥27 out of a maximum of 40).^62^ We also generated a binary variable for each individual Likert-scale stress indicator by grouping responses into high- vs low-score categories. We defined a high score as often/always reporting indicators that express a negative emotion (e.g., feeling angry), and never/rarely reporting indicators that express a positive emotion (e.g., feeling on top of things).^63^

#### Analysis Approach

We used generalized linear models to assess the relationship among the three water interruption definitions and each outcome variable. We used a Poisson error distribution with a log link for binary outcomes, a negative binomial error distribution with a log link for overdispersed count outcomes (number of times of antibiotic use), and a Gaussian error distribution with an identity link for continuous outcomes. Models adjusted for potential confounders, including sociodemographic factors (e.g., household wealth quintile, education), water, sanitation, and hygiene indicators (e.g., drinking water source, improved latrine access, handwashing station with soap), and animal ownership (Text S1). We did not control for water use and hygiene variables, as we hypothesized these to be on the causal pathway (Figure S1). We excluded binary and categorical confounders that did not sufficiently vary across our sample (<5% prevalence in any stratum). Models implemented robust standard errors to account for clustered outcomes. A cluster was defined as a geographic grouping of households ≥100 m distant from the next group.^64^ We used ArcGIS (ESRI, Redlands, California) to define clusters using the defined distance method based on the GPS coordinates of enrolled households. Analyses were conducted in R (version 4.3.1) RStudio (2023.06.0 + 421).

## Results and Discussion

### Participant Characteristics

We enrolled 237 households within 38 spatial clusters between June 20 and July 11, 2024. The mean age of respondents was 31.1 years, and households had on average 1.2 children <5 years (Table S1). Approximately 40% of respondents had some primary education. An average household spent $15–18 USD/week. Almost all households had cement/concrete floors, brick walls, and tin roofs. The most common fuel was charcoal, and the most common stove type was traditional solid fuel stoves (Table S1). Almost all households had a latrine, and approximately 20% had an improved latrine; the most common latrine type was a twin-pit latrine with a slab (Table S1). Most (84.4%) households had an improved primary drinking water source, including piped supplies (in their own dwelling, in their own yard/plot, or outside the compound) (56.6%) and boreholes (21.5%) (Table S1).

### Water Interruptions

Approximately one-third (32.5%, 77/237) of households reported at least one water interruption in the last 4 weeks. Specifically, 15.2% (36) of households experienced an interruption rarely (1– 2 times), 12.2% (29) sometimes (3–10 times), 3.8% (9) often (11–20 times), and 1.3% (3) always (>20 times) (Figure S2, Table S2). The mean duration of the last reported interruption was 4.53 days, and the median duration was 1.27 days (interquartile range: 1–3 days) (Figure S2, Table S2). Among households with interruptions, the cross-categories of interruption frequency and duration were: 25% (18) rare and short, 20% (15) rare and long, 25% (18) frequent and short, and 30% (22) frequent and long (Table S2). A subset (17%) of intermittencies occurred within a single geographic cluster; the remaining intermittencies were spread across other study clusters. Interruption patterns (frequency and duration) were similar across clusters.

Interruptions were mostly unpredictable: 13.0% (10) of households were notified in advance, 2.6% (2) could predict the interruption based on season or previous patterns, and 84.4% (65) were unaware of the upcoming interruption (Table S2). Interruptions were primarily associated with piped water supplies rather than wells; households with interruptions were more likely to obtain their primary drinking water from a piped connection outside the compound (39.0% vs 24.4%) and less likely to obtain it from a borehole (11.7 vs 26.3%) (Table S1). Households with vs without interruptions were broadly similar in their other characteristics (Table S1).

### Water Insecurity, Hygiene, and Water Use Practices

#### Water Insecurity

Among households with no water interruptions, the mean HWISE score was 1.9 out of 36, and 4.4% (7) of households were water-insecure (HWISE score ≥12) (Table 1). In adjusted analyses controlling for sociodemographic, water, sanitation, and hygiene indicators (Text S1), households with interruptions had higher HWISE scores (both for full and truncated score) and were 5 times more likely to be water-insecure (prevalence ratio [PR] = 5.34 (1.93, 14.8), *p* = 0.001) than those without interruptions (Table 1). Households with frequent interruptions had the highest mean HWISE scores and the highest likelihood (29.3%) of being water-insecure among all groups, while households with long vs short interruptions had similar mean scores (Table S3).

**Table 1:** Water Insecurity, Hygiene, and Water Use Practices by Households’ Water Interruption Status in the Last 4 Weeks^a^

	no interruption	**i**nterruption	interruption vs no interruption			
	*N* = 160	*N* = 77	unadjusted		adjusted	
			Δ/PR (95%CI)^c^	*p*-value	Δ/PR (95% Cl)^c^	*p*-value
						
**Water insecurity**						
the household water insecurity score, mean (SD)	1.9 (4.2)	8.1 (5.9)	**6.22 (4.78,7.65)**	**0.001**	**5.04(3.91,6.17)**	**0.001**
truncated household water insecurity score, mean (SD)^b^	1.9 (4.2)	6.4 (5.8)	**4.53 (3 08, 5.98)**	**0.001**	**3.36 (2.18, 4.55)**	**0.001**
the household is water-insecure, % (*n*)	4.4% (7)	20.8% (16)	**4.75 (2.37,9.52)**	**0.001**	**5.34 (1.93, 14.8)**	**0.001**
**Domestic and personal hygiene**						
the household has a handwashing station, % (*n*)	9.4% (15)	11.7% (9)	1.25 (0.68,2.30)	0.48	1.70(0.53,5.41)	0.38
the household has a handwashing station with water, % (*n*)	8.8% (14)	9.1% (7)	1.04 (0.50,2.17)	0.92	1.34 (0.38,4.77)	0.65
household members had to go without 3+ times in the last month, % (*n*)						
washing clothes	9.4% (15)	36.4% (28)	**3.88 (2.04,7.37)**	**0.001**	**3.56(2.04,6.21)**	**<.0005**
bathing	1.3% (2)	10.4% (8)	**8.31 (1.96,35.3)**	**0.001**	**13.40 (2.19, 82.6)**	**0.01**
washing hands after dirty activities	3.1% (5)	5.2% (4)	1.65 (0.62,4.45)	0.31	1.29 (0.29,5.74)	0 73
reported handwashing occasions, % (*n*)						
after defecation	90.0% (144)	94.8% (73)	1.05 (0.98, 1.14)	0.18	1.02(1.00,1.24)	0.05
after handling the child’s waste	63.1% (101)	61.0% (47)	0.97 (0.78, 1.21)	0.65	1.06 (0.89,1.27)	0.49
after handling domestic animals	10.0% (16)	3.9% (3)	0.39 (0.12, 1.25)	0.11	0.48 (0.21,1.08)	0.08
after handling animal feces	8.8% (14)	3.9% (3)	0.45 (0.15, 1.28)	0.13	**0.30 (0.12,0.75)**	**0.01**
after working (garden, market, etc.)	25.0% (40)	15.6% (12)	**0.62 (0.42,0.92)**	**0.02**	**0.58 (0.39, 0.86)**	**0.01**
after eating	81.3% (130)	79.2% (61)	0.98 (0.85, 1.11)	0.71	1.04 (0.89, 1.22)	0.59
before eating	91.9% (147)	94.8% (73)	1.03 (0.97, 1.09)	0 28	1.05 (0.99, 1.11)	0.12
before preparing food	09.4% (111)	62.3% (48)	0.90 (0.69, 1.17)	0.43	0.96 (0.77,1.20)	0.74
before feeding the child	41.3% (66)	42.9% (33)	1.04 (0.80, 1.35)	0.78	1.13(0.87.1.48)	0.37
before handling water (storage)	23.8% (38)	24.7% (19)	1.04 (0.69, 1.56)	0.85	1.29 (0.88, 1.87)	0.19
before breastfeeding	7.5% (12)	9.1% (7)	1.21 (0.54,2.71)	0.64	3.19(1.25, 8.17)	0.02
**Drinking water handling**						
water sample provided from storage container, % (*n*)	87.5% (140)	96.1% (74)	**1.08 (1.00,1.17)**	**0.04**	1.08(1.00, 1.17)	0.05
stored water container fully or partially covered, % (*n*)	94.0% (126)	89.9% (62)	0.96 (0.88, 1.04)	0.27	0.89(0.81,0.96)	0.01
stored water container has a narrow mouth, % (*n*)	17.5% (28)	5.2% (4)	**0.28 (0.16,0.49)**	**0.001**	**0.18 (0.09, 0.39)**	**<.0005**
water sample reported to be treated, % (*n*)	5.6% (9)	2.6% (2)	0.46 (0.09,2.30)	0.34	0.44(0.05,3.79)	0.46
duration of water storage in hours, mean (SD)	32.0 (54.8)	29.9 (37.3)	–2.73 (−15.6,10.1)	0.68	–11.7 (−30.2, 7.26)	0.23
primary drinking water source feels acceptable, % (*n*)	89.4% (143)	84.4% (65)	0.94 (0.85, 1.05)	0.29	1.00 (0.90,1.08)	0.78
the household obtains drinking water from a secondary source, % (*n*)	24.4% (39)	32.5% (25)	1.33 (0.83,2.14)	0.24	1.25 (0.71,2.19)	0.45
secondary drinking water source is improved, % (*n*)	36.8% (14)	53.9% (14)	1.46 (0.77,2.76)	0.24	**2.90 (1.26, 6.70)**	**0.01**
the household obtains nondrinking water from an improved source, % (*n*)	65.6% (105)	64.9% (50)	0.99 (0.78, 1.25)	0.93	1.02 (0.82,1.25)	0.89

a Bolded estimates indicate statistically significant associations at *p* < 0.05 level.

b The truncated household water insecurity (HWISE) score excludes the intermittency question.

c Δ: Difference in means for continuous outcome variables. PR: prevalence ratio for binary outcome variables; CI: confidence interval; SD: standard deviation.

#### Domestic and Personal Hygiene

Households with vs without water interruptions reported similar access to water for handwashing; approximately 9% of households in both groups had a handwashing station with available water observed by enumerators (Table 1). Households with rare vs frequent interruptions had similar access to a handwashing station with water, while households with short interruptions had the lowest access (2.8%) among all groups (Table S3).

Water interruptions were associated with restrictions in some but not all of the hand hygiene behaviors. Respondents with vs without interruptions reported similar handwashing prevalence after defecating or handling child feces, and before eating or handling food or water (Table 1). However, households with interruptions were 3 times less likely to report handwashing after handling animal feces (PR = 0.30 (0.12–0.75), *p* = 0.01), and half as likely to report handwashing after working outside (e.g., garden, market) (PR = 0.58 (0.39, 0.86), *p* = 0.01) than households without interruptions (Table 1). Among households with long interruptions, none reported handwashing after handling domestic animals or animal feces (Table S3). Water interruptions were also associated with impaired domestic hygiene, measured in individual HWISE questions. Households with interruptions were 3 times more likely to go without laundry (PR = 3.56 (2.04–6.21), *p* < 0.0005) and >10 times more likely to go without bathing (PR = 13.4 (2.19–82.6), *p* = 0.01) at least 3 times in the last 4 weeks (Table 1). Households with frequent interruptions were more likely to go without laundry and bathing than other groups (Table S3).

#### Drinking Water Handling

When asked to provide a glass of drinking water the same way they would serve a young child, most respondents obtained water from storage containers regardless of water interruptions (96.1% of households with interruptions, 87.5% of households without interruptions; Table 1). Most (≥90%) storage containers in both groups were observed to be covered, but only 15% of containers had a narrow mouth; households with interruptions were less likely to use narrow-mouthed containers (PR = 0.18 (0.09–0.39), *p* < 0.0005) (Table 1). Among households that provided drinking water from storage, the water had been collected approximately 1.5 days ago on average (Table 1). Drinking water treatment was rare in both groups. Households with vs without interruptions were similarly likely to rate their primary drinking water source acceptable and use secondary drinking water sources (Table 1). Among households with rare and short interruptions, all provided drinking water samples from storage containers; they were also more likely than other groups to use a secondary drinking water source (Table S3). Households with frequent interruptions were the least likely to rate their water source as acceptable (Table S3).

### Water Quality

Of 236 drinking water samples, 65.7% (155) harbored *E. coli* (geometric mean = 0.74 log10-MPN/100 mL, arithmetic mean = 98.1 MPN/100 mL). Of 226 samples processed with cefotaxime supplementation, 8.4% (19) harbored cefotaxime-resistant *E. coli* (geometric mean = −0.23 log10-MPN/100 mL, arithmetic mean = 1.3 MPN/100 mL). Among the 19 positive samples, the relative abundance of cefotaxime resistance (ratio of cefotaxime-resistant to generic *E. coli* counts for the same sample) ranged from 0.1 to 70%. Water source types with the highest mean levels of contamination ranged from 866.4 MPN/100 mL for protected spring (*n* = 1), 217.3 MPN/100 mL for boreholes (*n* = 51), 135.6 MPN/100 mL for piped water into the dwelling (*n* = 20), and 81.3 MPN/100 mL for piped water outside the compound (*n* = 69) (Table S4).

In adjusted analyses controlling for sociodemographic, water, sanitation, and hygiene indicators (Text S1), households with interruptions had similar prevalence and counts of both generic and cefotaxime-resistant *E. coli* (Table S5). There were no significant differences in *E. coli* prevalence or counts between categories of interruption frequency (Table S6) or duration (Table S7). We could not assess associations between these categorical variables and cefotaxime-resistant *E. coli* because of the small number of positive samples.

### Child Health

Among children not experiencing water interruptions, 16.3% had caregiver-defined diarrhea, 13.9% had WHO-defined diarrhea, 40.6% had ARI, and 17.3% had ARI with fever in the last 7 days, while 29.7% of children were reported to have taken antibiotics at least once in the last 4 weeks (Table S5).

#### Interruption Occurrence

In adjusted analyses, children experiencing water interruptions had higher prevalence of both caregiver-defined diarrhea (prevalence ratio [PR] = 1.85 (1.02–3.37), *p* = 0.04) and WHO-defined diarrhea (PR = 1.53 (0.72–3.25), *p* = 0.27) but the association for WHO-defined diarrhea could not be distinguished from chance (Figure 1, Table S5). Children experiencing interruptions also had 2 times higher prevalence of ARI with fever (PR = 1.98 (1.09–3.57), *p* = 0.02) (Figure 1, Table S5). There were no significant associations between water interruptions and antibiotic use in children (Figure 1, Table S5). Among our negative control outcomes, interruptions were not associated with the prevalence of having a rash (PR = 1.32 (0.64–2.73), *p* = 0.45) (Table S5). We could not assess associations for our second negative control outcome because only 2.4% (7) of children were reported to have ear infections.

**Figure 1:**
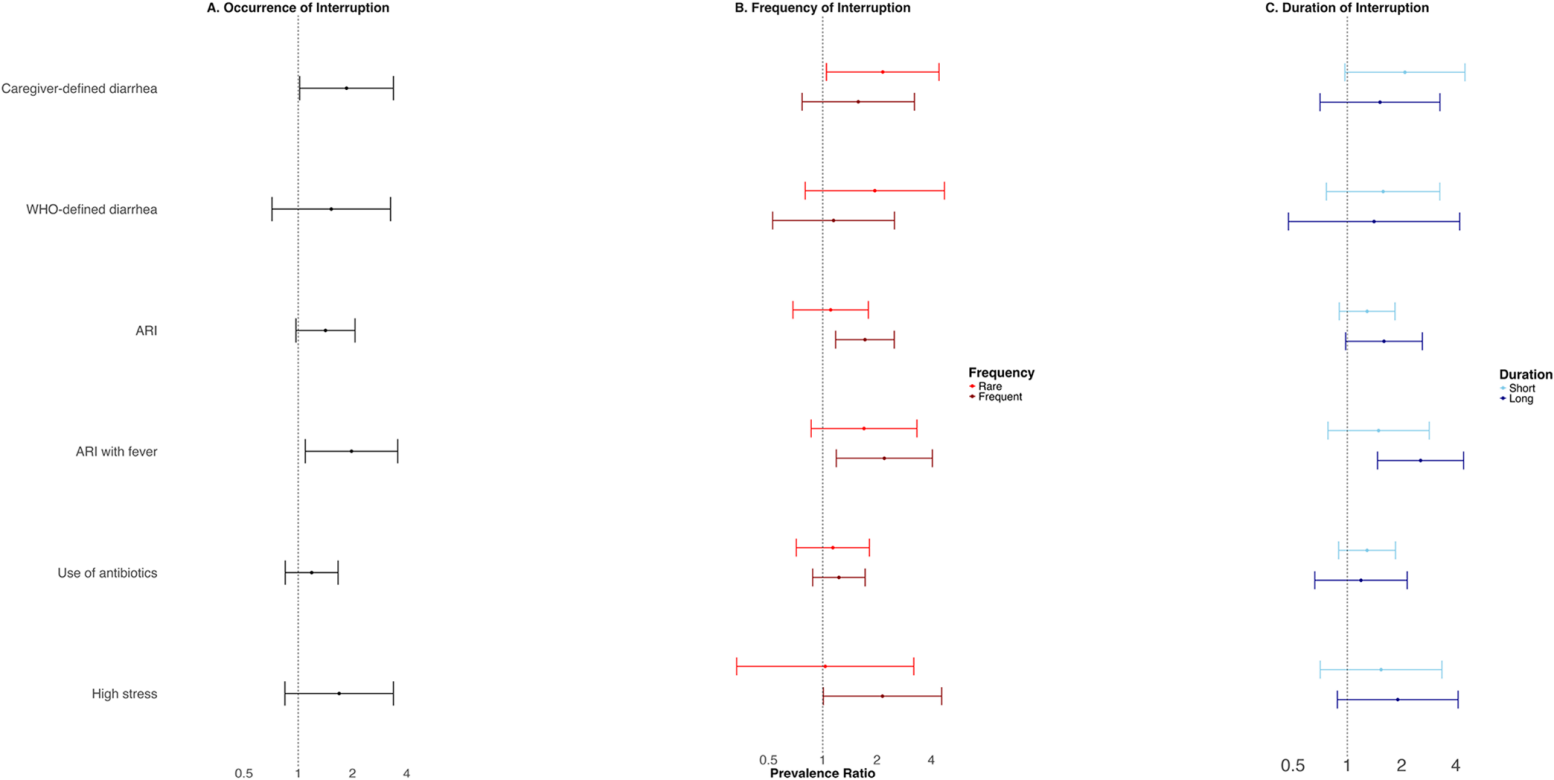
Adjusted associations between caregiver-reported child health and caregiver stress outcomes and (A) binary occurrence of water interruption in the last month, (B) categorical frequency of interruption in the last month (none, rare, frequent), and (C) categorical duration of the last experienced interruption (none, short, long). Rare refers to 1–2 interruptions, frequent refers to 3+ interruptions in the last month. Short refers to below-median interruption duration (<1.3 days), long refers to above-median interruption duration (≥1.3 days). Households with no interruptions are the reference group in all models. The circles denote point estimates for prevalence ratios, and the horizontal lines denote 95% confidence intervals. Models controlled for age of the respondent, age of the target child, total number of individuals living in household, respondent’s highest level of education, highest level of education level for anyone in household, asset-based household wealth quintile, average weekly expenditures by household, household food insecurity score, whether household used piped water for their primary water source, whether household’s designated handwashing station had soap, whether household had an improved latrine (as defined by the JMP), flooring material inside household, total number of animals in compound, and respondent’s report of the last time it rained.

#### Interruption Frequency

Children experiencing rare interruptions (1–2 in last month) had 2 times higher prevalence of caregiver-defined diarrhea (PR = 2.15 (1.05–4.42), *p* = 0.04) and WHO-defined diarrhea (PR = 1.95 (0.80, 4.73), *p* = 0.14) and used antibiotics twice as many times (count ratio [CR] = 1.98 (1.04–3.78), *p* = 0.04) compared to children not experiencing interruptions, but the association for WHO-defined diarrhea could not be distinguished from chance (Figure 1, Table S6). Children experiencing frequent interruptions (3+ in last month) had approximately 2 times higher prevalence of ARI (PR = 1.72 (1.18–2.49), *p* = 0.005) and ARI with fever (PR = 2.20 (1.19–4.06), *p* = 0.01) than those not experiencing interruptions; there were no associations with other child health outcomes (Figure 1, Table S6).

#### Interruption Duration

Children experiencing short interruptions (<1.27 days) had 2 times higher prevalence of caregiver-defined diarrhea (PR = 2.09 (0.97–4.49), *p* = 0.06) and appeared to have a higher number of antibiotic use episodes than children not experiencing interruptions, but these associations could not be distinguished from chance (Figure 1, Table S7). Children experiencing long interruptions (≥1.27 days) had higher prevalence of ARI (PR = 1.60 (0.98–2.61), *p* = 0.06) and ARI with fever (PR = 2.55 (1.47–2.85), *p* < 0.0005) than those not experiencing interruptions, but the association with ARI could be distinguished from chance; there were no associations with other child health outcomes (Figure 1, Table S7).

### Caregiver Stress

Among caregivers not experiencing water interruptions, the mean PSS score was 18.8 out of 40, and 9.4% (15) of caregivers experienced high stress (PSS score ≥27) (Table S5).

#### Interruption Occurrence

Caregivers experiencing water interruptions had higher PSS composite scores and were twice as likely to experience high stress in unadjusted analyses, but these associations could not be distinguished from chance after adjusting for confounders (Figures 1, S3 and Table S5). In adjusted analyses, caregivers experiencing interruptions were more likely to report feeling unable to control things (PR = 1.69 (1.01–2.83), *p* = 0.05) or angry (PR = 1.75 (0.98, 3.14), *p* = 0.06), though the latter association could not be distinguished from chance (Figure S3, Table S5). There were no associations between water interruptions and other stress indicators.

#### Interruption Frequency

Caregivers experiencing rare interruptions had similar PSS scores and likelihood of experiencing high stress as those not experiencing interruptions (Figures 1, S3 and Table S6). They were twice as likely to report feeling angry (PR = 2.27 (1.15, 4.49), *p* = 0.02) (Table S5). Caregivers experiencing frequent interruptions had higher composite PSS scores (ΔPSS = 2.06 (0.24–3.89), *p* = 0.03) and were twice as likely to experience high stress (PR = 2.14, (1.01–4.47), *p* = 0.05) compared to caregivers not experiencing interruptions (Figures 1, S3, Table S6).

#### Interruption Duration

Caregivers experiencing short interruptions had higher PSS scores than those not experiencing interruptions (ΔPSS = 1.80 (0.19–3.42), *p* = 0.03) (Table S7). They were also 2 times more likely to feel unable to control things (PR = 1.90 (1.08, 3.35), *p* = 0.03), nervous or stressed (PR = 1.89 (1.03–3.49), *p* = 0.04), and angry (PR = 2.21 (1.12, 4.36), *p* = 0.02) (Figure S3, Table S7). There were no significant associations between long interruptions and stress indicators (Figure 1, Table S7).

## Discussion

One-third of households in our study experienced at least one water supply interruption in the last 4 weeks. Households experiencing interruptions were more water-insecure and more likely to deprioritize some hygiene practices, especially around animal management, laundry, and bathing. Most (>90%) households relied on stored drinking water, while drinking water quality, as measured by the presence and abundance of *E. coli*, was similar between the groups. Children experiencing interruptions had a higher prevalence of caregiver-defined diarrhea and ARI with fever; rare interruptions were associated with diarrhea, and frequent or long interruptions were associated with ARI. Caregivers experiencing short or frequent interruptions reported higher stress.

Notably, households in our study experienced sporadic interruptions of a continuous supply rather than systematic intermittent supply (e.g., rotating water service). Only about 15% of participants experienced an interruption more than twice in the last month, and the median duration of the last reported interruption was approximately 1 day. This differs from studies of intermittently operating systems, where water may be delivered for a set number of hours once every few days,^65^ and more closely aligned with studies of water supply interruptions in otherwise continuous systems.^66^ Importantly, both sporadic interruptions and systemic intermittencies have been associated with adverse health outcomes.^11^

Water intermittency is an important component of water insecurity. Household water insecurity scores were low in our study, and less than 10% of households were water-insecure. Despite being relatively water-secure, households that experienced at least one water interruption in the last 4 weeks were substantially more likely to skip some hygiene practices during the same period. Importantly, households with and without interruptions reported similar handwashing during some key moments (after defecation, before eating or handling food or water), consistent with evidence that washing hands after defecation is prioritized despite water limitations.^67,68^ However, households with interruptions in our study reported substantially lower handwashing after handling animal feces or working outside (e.g., in the garden) and were more likely to skip laundry and bathing. Unsafe handling of domestic animals is a risk factor for infectious diseases.^69–71^ Domestic soil is also increasingly recognized as a disease transmission pathway in settings where fecal waste is not isolated from the environment.^72,73^ Given that the majority (75%) of households in our study had animals in their compound, water conservation strategies triggered by water supply interruptions may potentially increase the transmission of zoonotic and soilborne pathogens. Identifying and addressing the specific hygiene behaviors most affected by water interruptions can support effective strategies to mitigate health impacts.

Water interruptions can force households to rely on stored drinking water, a well-documented risk factor for microbial contamination.^15^ In our study, the majority of households provided drinking water from storage regardless of interruption status, likely reflecting a lack of on-premise plumbing. *E. coli* prevalence or counts did not differ between households with vs without interruptions, in contrast with previous studies.^14,15,74^ There could be several reasons for this finding. First, storing water prior to use attenuates water quality differences between intermittent and continuous supplies by introducing contamination during storage and handling.^65^ Second, we did not collect water samples during or immediately after interruptions. At the time of sample collection, households reported at least one interruption in the last 4 weeks, but we did not record how long ago the last interruption occurred. Therefore, our samples may not reflect the conditions (water source or quality) during or shortly after the interruption. Further, interruptions more commonly occurred in piped water sources; households with interruptions were more likely to obtain their primary drinking water from a piped connection outside the compound and less likely to obtain it from a borehole. While both of these sources are classified as improved by the JMP, piped connections outside the compound had lower *E. coli* levels (mean = 81 MPN/100 mL) than boreholes (mean = 217 MPN/100 mL) in our study. Our analyses controlled for whether the household’s drinking water came from a piped source, but our findings of no adverse water quality effects from interruptions may residually reflect the better quality of piped sources, which more commonly experienced interruptions. This trend is consistent with JMP data that piped supplies are less likely than nonpiped supplies to meet the Sustainable Development Goal criterion of being “available when needed.”^75^ Importantly, our findings highlight a trade-off between water supply reliability and quality, with boreholes presenting a supply that is less likely to be interrupted but more likely to be contaminated than piped sources. In this setting, borehole water treated at the point-of-use may provide a safe alternative during piped water interruptions.

Children experiencing water supply interruptions had increased diarrhea, consistent with prior evidence.^5,36,65^ Our study also demonstrated a novel link between water interruptions and respiratory infections. Interruptions may increase ARI through compromised hygiene, consistent with impaired handwashing among households experiencing interruptions in our study. Diarrhea episodes have also been shown to increase the risk of subsequent respiratory infections.^76^ A small number of studies to date have shown reductions in ARI from hygiene and water quality improvements.^21,24,77^ Emerging research suggests that water intermittencies can hamper adherence to handwashing recommendations for COVID-19 prevention and increase COVID-19 infections.^25–27^ While ARIs are responsible for a major morbidity and mortality burden among young children in low-income countries,^78^ existing disease burden estimates for intermittent water supplies are focused on enteric infections only.^5^ Our findings indicate that these assessments may underestimate the total disease burden from water supply interruptions.

Increasing interruption frequency and duration affected health outcomes differentially in our study rather than following a dose–response pattern. Children experiencing rare interruptions had higher diarrhea prevalence, while children with frequent or long interruptions had higher ARI prevalence. These findings suggest distinct mechanisms through which interruption frequency and duration impact risk factors for enteric versus respiratory pathogen transmission. For example, rare or short interruptions may catch households unprepared and acutely disrupt hygiene, while frequent or long interruptions may trigger sustained adaptive changes, such as water rationing, that introduce unintended direct or indirect exposure pathways. Efforts to mitigate the health impacts of water supply interruptions should consider interruption patterns to design and implement effective interventions. Our findings on hygiene practices and water quality also suggest that, in our context, the increased risk of enteric and respiratory infections associated with water interruptions may be more strongly driven by the observed changes in hygiene behaviors from limited water quantity than by differences in water quality.

Almost one-third of children <5 years in our study used antibiotics in the last 4 weeks. Children experiencing rare interruptions had higher antibiotic use than those not experiencing interruptions; this interruption pattern was also associated with a higher prevalence of caregiver-defined diarrhea, which may prompt care-seeking and antibiotic use. Antibiotics are heavily used to treat diarrheal and respiratory infections among children in low-income countries, including sub-Saharan Africa.^51,52^ Frequent antibiotic exposure can trigger antimicrobial resistance among the gut flora^79^ and pathogens.^80^ Our findings suggest that increased diarrhea among children experiencing some intermittency patterns may translate to increased antibiotic use and potentially antimicrobial resistance. If interrupted water supplies are more heavily contaminated with antimicrobial-resistant bacteria, ingestion of drinking water can also increase the risk of gut colonization with these organisms.^81^ A study in India detected cefotaxime resistance in 57% of *E. coli* isolates from an intermittent piped water system.^18^ In our study, 8% of drinking water samples harbored cefotaxime-resistant *E. coli*, suggesting some ingestion of antimicrobial-resistant bacteria via drinking water. However, the prevalence and abundance of cefotaxime-resistant *E. coli* did not differ between households with and without water interruptions. Our findings suggest other potential pathways from water interruptions to antimicrobial resistance (e.g., impaired hygiene leading to enteric/respiratory infections leading to antibiotic use) that merit future investigation.

Our findings align with prior research linking water insecurity to increased psychosocial distress, particularly among caregivers responsible for household water management.^8,31,34,35,38,42^ We found that shorter and more frequent water supply interruptions were associated with higher stress, which may be due to greater uncertainty and the need for quick adaptation strategies, suggesting that even brief disruptions in water access can have psychological effects. A recent study found that being able to predict a water interruption can reduce this stress response.^13^ A review found that unpredictable intermittencies, compared to regular and predictable intermittencies, were the most disruptive in the lives of consumers across >100 case studies.^6^ We were not able to test the influence of predictability on stress because most households reported that they were not notified about the interruption or able to predict it. Our findings reinforce calls for integrating mental health considerations into water supply programs.

Climate change is expected to exacerbate water access challenges by increasing the frequency and severity of droughts, rainfall variability, and extreme weather events. These stressors can disrupt traditional water sources, potentially increasing reliance on unprotected sources, overcrowding and social conflict around water access. Additionally, climate change can alter seasonality, stressing households as protected, piped water may be less available during dry months, while unprotected sources such as surface water may be more accessible during rainy seasons.^41,82^ Previous studies have documented seasonal shifts in intermittency patterns and water quality effects.^65^ Our study was conducted during Malawi’s dry season when water interruptions may occur more frequently, and alternative sources may be less available. Interruption patterns and their relationships with our study outcomes may be different during the rainy season. As changing weather patterns, extreme events, and shifting seasonal cycles increasingly disrupt water availability, future research should quantify health and well-being impacts from increasingly unpredictable and intermittent water supplies.^16^

Water access inequities often align with wealth disparities, with lower-income households relying on communal sources disproportionately affected.^8,83^ In our study, households with and without interruptions were socioeconomically similar, suggesting additional drivers of water supply interruptions, such as geographic and infrastructural challenges.^83–85^ Kumar et al. found in Bengaluru, India, that low-income and disadvantaged areas received water more frequently and predictably than wealthier areas, suggesting service quality does not always reflect socioeconomic status.^86^ While we did not assess why the observed interruptions occurred, water utilities in our study setting report a complex set of causes, including compromised infrastructure, power shortages that disrupt pumping, as well as water shortages and high demand during dry seasons.^87^ Aging infrastructure and widespread leakages account for almost 40% of physical water losses, and demand regularly exceeds supply capacity, especially during dry periods.^50^ High levels of nonrevenue water—exceeding 50%—due to illegal connections, faulty meters, and system leaks further strain utilities’ resources.^88^ Additionally, water access is highly unequal across zones, with peri-urban and elevated areas experiencing especially short and inconsistent supply due to low pressure and distribution issues.^50,88^ Urban planning and infrastructure investment decisions should prioritize maintaining distribution system pipes (e.g., minimize leaks) and reinforcing power supplies (e.g., prevent pumping disruptions) to reduce interruptions in piped water systems while expanding access to source water supplies to meet demand.

Our study integrated microbial, behavioral, health, and psychosocial dimensions of water interruptions for a comprehensive assessment. This study also had limitations. Both water interruptions and outcomes were self-reported and may be subject to recall bias. Households experiencing interruptions may be aware of health risks and overreport adverse health outcomes. However, the lack of associations between interruptions and our negative control outcome (rash) suggests that recall bias is unlikely to explain the findings. We also note that while we assumed that rashes are causally independent of water interruptions, hygiene behaviors could influence skin conditions. Further, our observational analysis limits causal inference, and residual confounding may remain from unaccounted-for differences between households with vs without interruptions. Another limitation is that our exposures and outcomes were measured concurrently and had different recall periods (last 4 weeks for water interruptions, antibiotic use, and caregiver stress, last 7 days for child diarrhea and ARI); therefore, we could not establish exposure-outcome temporality, but it is unlikely that reverse causation (i.e., outcomes causing the exposure) explains our findings. Additionally, we relied on the duration of the last interruption for analysis; this may not be representative of the typical interruption duration. Finally, we did not correct for multiple hypothesis testing because Bonferroni corrections and other multiplicity adjustments can lead to overcorrections when outcomes are correlated.^89^ Therefore, some reported associations could be due to chance, but findings were consistent across different analyses, and exposure-mediator associations aligned with exposure-outcome associations. Therefore, we conclude that our findings are internally consistent, biologically plausible, and unlikely to be explained by chance.

In summary, our findings indicate that water supply interruptions are associated with impaired hygiene (especially around handling domestic animals), higher risk of enteric and respiratory infections in children, and increased stress among caregivers, with differential effects from different patterns of interruption frequency and duration. Future studies should evaluate whether specific coping strategies (e.g., low-flow hand hygiene strategies, water reuse for domestic hygiene, advanced notification on upcoming disruptions) may help reduce physical and mental health risks during water supply interruptions, while provision of an uninterrupted continuous water supply remains the ultimate goal.

## Supplementary Material



## Notes

Directed acyclic graph of hypothesized causal pathways (Figure S2); adjusted model covariates (Text S1); sociodemographic, water and sanitation indicators (Table S1); water interruption characteristics (Table S2 and Figure S2); water use practices (Table S3); water quality by source type (Table S4); outcomes by interruption occurrence, frequency and duration (Tables S4–S7 and Figure S3) (PDF)
