# Supplementary material for: Associations between Water Supply Interruptions and Water Use, Drinking Water Quality, Child Health, and Caregiver Stress in Peri-Urban Malawi
Source: Environ Sci Technol. 2026 Jan 15;60(4):2917–29. doi: 10.1021/acs.est.5c08820 (PMC12874536; doi:10.1021/acs.est.5c08820)
Supplement: Supplementary file 1 [file es5c08820_si_001.pdf]

Supporting information

Associations between water supply interruptions and water use, drinking water quality, child health, and caregiver stress in peri-urban Malawi

Caitlin G Niven, Benjamin Clark, Emily Floess, Blessings Chirwa, Monica Matekenya, Emma Budden, Stella Cadono, John Chavula, Victor Chisamanga, Aubrey Dzinkambani, Chisomo Kaponda, Neema Ngondo, Norah Patterson, Sheena Symon, Brighton A Chunga, Rochelle H Holm, Petros Chigwechokha, Francis L. de los Reyes III, Cassandra L Workman, Angela R Harris, Ayse Ercumen

Table of Contents

Figure S1. Directed acyclic graph, page S1

Text S1. Adjusted model covariates, page S1

Table S1. Socio-demographics and water/sanitation indicators among study households, page S2

Table S2. Water supply interruption characteristics, page S3

Figure S2. Histogram of (a) interruption frequency and (b) duration of last interruption, page S4

Table S3. Water use practices by interruption frequency and duration, page S5

Table S4. Water quality by water source type, page S6

Table S5. Study outcomes by binary interruption occurrence, page S7

Table S6. Study outcomes by categorical interruption frequency, page S8

Table S7. Study outcomes by categorical interruption duration, page S9

Figure S3. Associations between caregiver stress and water interruption (a) occurrence, (b) frequency, and (c) duration, page S10

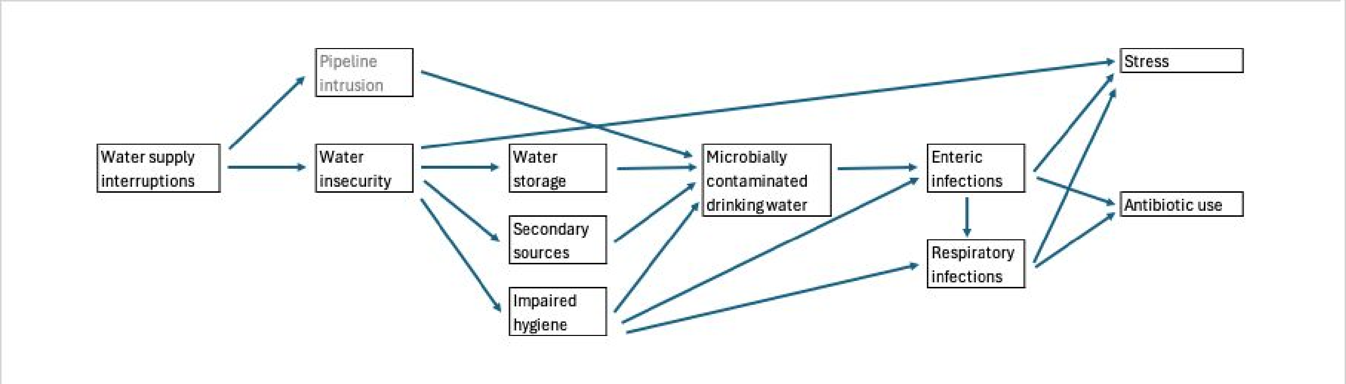

Figure S1. Directed acyclic graph illustrating the hypothesized causal pathways between water supply interruptions, mediating behaviors and our measured outcomes.

Text S1. Adjusted model covariates.

All models controlled for:

- Household's primary water source is piped
- Household has an improved latrine
- Household has handwashing station with soap (observed)
- Number of animals living on the compound
- Age of respondent
- Total number of people living in the household
- Respondent's highest level of education
- Highest education level in the household
- Household wealth index quintile
- Amount of money spent per week by the household, in USD
- Floor material in the household
- Respondent's report of last time it rained

Models for water quality outcomes additionally controlled for:

- Water sample is obtained from piped source
- Water sample is reported to be treated

Models for child health and caregiver stress outcomes additionally controlled for:

- Child age, in months
- Food insecurity index (HFIAS score)

We considered but did not control for the following covariates because they had <5% variation in the study sample (i.e. one category dominated across all households):

- Roof materials
- Wall materials
- For respiratory outcomes (as predictors of indoor air quality):
  - Fuel type
  - Stove type
  - Presence of windows in dwelling

Table S1. Socio-demographic and water and sanitation indicators among study households

	No Interruption N=160	Interruption N=77
**Demographics**		
Respondent's age, mean (SD)	31.1 (10.5)	31.1 (9.8)
Number of individuals living in household, mean (SD)		
Children <5 years	1.2 (0.5)	1.2 (0.4)
Children 5-16 years	1.4 (1.3)	1.3 (1.1)
Adults >16 years	2.7 (1.2)	2.8 (1.5)
**Socio-economic indicators**		
Respondent's highest level of education, % (n)		
Incomplete primary	24.4% (39)	35.1% (27)
Complete primary	19.4% (31)	5.2% (4)
Incomplete secondary	25.0% (40)	28.57% (22)
Complete secondary	24.4% (39)	22.1% (17)
Post-secondary	5.0% (8)	7.8% (6)
Highest level of education by any household member, % (n)		
Incomplete primary	9.6% (15)	15.6% (12)
Complete primary	6.4% (10)	5.2% (4)
Incomplete secondary	20.4% (32)	22.1% (17)
Complete secondary	45.9% (72)	36.4% (28)
Post-secondary	17.2% (27)	20.8% (16)
Household owns, % (n)		
Mobile phone	86.3% (138)	87.0% (67)
Television	41.9% (67)	49.4% (38)
Refrigerator	20.6% (33)	29.9% (23)
Mosquito net	73.1% (117)	63.6% (49)
Asset-based wealth quintiles, % (n)		
Bottom quintile	19.4% (31)	16.9% (13)
2nd quintile	21.3% (34)	18.2% (14)
3rd quintile	18.1% (29)	22.1% (17)
4th quintile	23.8% (38)	19.5% (15)
Top quintile	17.5% (28)	23.4% (18)
Money spent per week by household in USD, mean (SD)	18.2 (15.9)	14.7 (14.5)
**Animal presence**		
Household owns animals, % (n)	29.4% (47)	13.0% (10)
Animals live on the compound, % (n)	75.6% (121)	72.7% (56)
Number of animals owned by household, mean (SD)	2.0 (5.0)	1.6 (8.4)
Number of animals living on the compound, mean (SD)	6.1 (6.38)	9.2 (11.87)
**Housing characteristics, %(n)**		
Floors made out of cement/concrete	88.1% (141)	88.3% (68)
Walls made out of brick	89.4% (143)	94.8% (73)
Roof made out of tin	97.5% (156)	97.4% (75)
Dwelling has windows	95.0% (152)	98.7% (76)
Household uses charcoal (made from wood, traditional) for any purpose	95.0% (152)	94.8% (73)
Household uses traditional solid fuel stove (non-manufactured)	95.0% (152)	94.8% (73)
**Water access**		
Primary drinking water from improved source, % (n)	85.6% (137)	81.8% (63)
Type of water source, % (n)		
Tubewell/ borehole	26.3% (42)	11.7% (9)
Protected spring (has concrete lining)	0% (0)	1.3% (1)
Unprotected dug well	1.3% (2)	0% (0)
Protected dug well (has concrete lining)	6.9% (11)	3.9% (3)
Surface water (river, dam, lake, pond, stream, canal, channel)	0% (0)	2.6% (2)
Piped water into dwelling	7.5% (12)	10.4% (8)
Piped water into yard/plot	20.6% (33)	15.6% (12)
Piped water outside the compound	24.4% (39)	39.0% (30)
Vendor water (kiosk)	12.5% (20)	13.0% (10)
**Sanitation access**		
Household has latrine, % (n)	98.8% (158)	98.7% (76)
Household has improved latrine, % (n)	19.6% (31)	19.7% (15)
Households using a shared latrine, % (n)	70.3% (111)	75.0% (57)
Type of latrine, % (n)		
Flush/pour flush	1.9% (3)	0% (0)
Flush to piped sewer system	0% (0)	1.3% (1)
Flush to septic tank	8.9% (14)	4.0% (3)
Flush to pit latrine	0.6% (1)	1.3% (1)
Pit latrine without slab/open pit	0.6% (1)	0% (0)
Twin pit with slab	67.1% (106)	63.2% (48)
Twin pit without slab	26.0% (41)	32.9% (25)

a
b
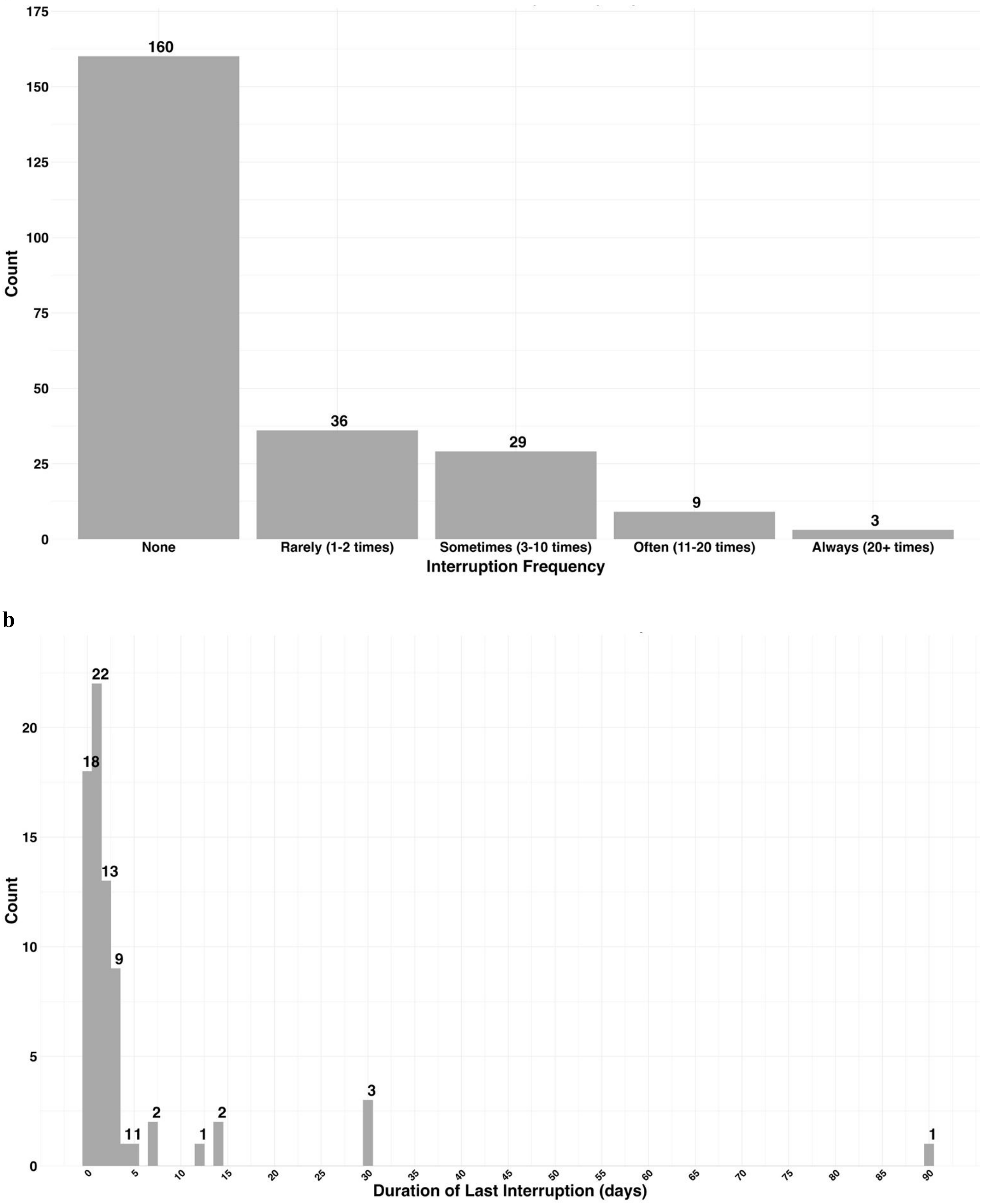

Fig S2. Histogram of (a) interruption frequency and (B) duration of last interruption.

Table S2. Water supply interruption characteristics

Interruption frequency, % (n)	N=237
Any interruption	32.5% (77)
Rare (1-2 times)	15.2% (36)
Sometimes (3-10 times)	12.2% (29)
Often (11-20 times)	3.8% (9)
Always (20+ times)	1.3% (3)
**Duration of last interruption, (days)**	N=73[a]
Mean (SD)	4.53 (11.96)
Median (IQR)	1.27 (1.00-3.00)
Range (min-max)	0.02-90.3
**Cross-categories of interruption frequency and duration**	N=73[a]
Rare (1-2 times) and short (<1.27 days)	25% (18)
Rare (1-2 times) and long (≥1.27 days)	20% (15)
Frequent (3+ times) and short (<1.27 days)	25% (18)
Frequent (3+ times) and long (≥1.27 days)	30% (22)
**Predictability of interruptions**	N=77
Notified in advance	13% (10)
Could predict based on season or pattern	3% (2)
Unpredictable	84% (65)

SD: Standard deviation; IQR: Interquartile range (25th-75th percentile)
[a] 4 households had missing data on interruption duration.

Table S3. Water use practices by interruption frequency and duration. Rare refers to 1-2 interruptions, frequent refers to 3+ interruptions in the last month. Short refers to below-median interruption duration (<1.3 days), long refers to above-median interruption duration (≥1.3 days).

	No Interruption	Interruption Frequency		Interruption Duration[a]	
		Rare	Frequent	Short	Long
	N=160	N=36	N=41	N=36	N=37
**Water insecurity**					
Household water insecurity score, mean (SD)	1.9 (4.2)	6.6 (5.2)	9.4 (6.3)	8.3 (6.8)	7.9 (5.2)
Truncated household water insecurity score, mean (SD) [b]	1.9 (4.2)	5.6 (5.2)	7.1 (6.3)	6.6 (6.7)	6.2 (5.1)
Household is water insecure, % (n)	4.4% (7)	11.1% (4)	29.3% (12)	22.2% (8)	16.2% (6)
**Domestic and personal hygiene**					
Household has handwashing station, % (n)	9.4% (15)	11.1% (4)	12.2% (5)	5.6% (2)	16.2% (6)
Household has handwashing station with water, % (n)	8.8% (14)	8.3% (3)	9.8% (4)	2.8% (1)	13.5% (5)
Household members had to go without 3+ times in last month, % (n)					
Washing clothes	9.4% (15)	30.6% (11)	41.5% (17)	33.3% (12)	37.8% (14)
Bathing	1.3% (2)	5.6% (2)	14.6% (6)	11.1% (4)	10.8% (4)
Washing hands after dirty activities	3.1% (5)	2.8% (1)	7.3% (3)	0% (0)	8.1% (3)
Reported handwashing occasions, % (n)					
After defecation	90% (144)	100% (36)	90.2% (37)	91.7% (33)	97.3% (36)
After handling child's waste	63.1% (101)	47.2% (17)	73.2% (30)	63.9% (23)	56.8% (21)
After handling domestic animals	10% (16)	2.8% (1)	4.9% (2)	8.3% (3)	0% (0)
After handling animal feces	8.8% (14)	2.8% (1)	4.9% (2)	8.3% (3)	0% (0)
After working (garden, market, etc.)	25% (40)	11.1% (4)	19.5% (8)	27.8% (10)	5.4% (2)
After eating	81.3% (130)	91.7% (33)	68.3% (28)	69.4% (25)	86.5% (32)
Before eating	91.9% (147)	100% (36)	90.2% (37)	97.2% (35)	91.9% (34)
Before preparing food	69.4% (111)	69.4% (25)	56.1% (23)	58.3% (21)	62.2% (23)
Before feeding child	41.3% (66)	33.3% (12)	51.2% (21)	44.4% (16)	43.2% (16)
Before handling water (storage)	23.8% (38)	19.4% (7)	29.3% (12)	44.4% (16)	8.1% (3)
Before breastfeeding	7.5% (12)	2.8% (1)	14.6% (6)	11.1% (4)	5.4% (2)
**Drinking water handling**					
Water sample provided from storage container, % (n)	87.5% (140)	100% (36)	92.7% (38)	100% (36)	91.9% (34)
Stored water container fully or partially covered, % (n)	78.8% (126)	83.3% (30)	78.0% (32)	88.9% (32)	75.7% (28)
Stored water container has narrow mouth, % (n)	17.5% (28)	11.1% (4)	0% (0)	2.8% (1)	8.1% (3)
Water sample reported to be treated, % (n)	5.6% (9)	5.6% (2)	0% (0)	2.8% (1)	2.7% (1)
Duration of water storage in hours, mean (SD)	32.6 (54.8)	36.8 (47.4)	23.3 (23.1)	35.1 (44.9)	25.6 (29)
Primary drinking water source feels acceptable, % (n)	89.4% (143)	91.7% (33)	78.0% (32)	80.6% (29)	89.2% (33)
Household obtains drinking water from secondary source, % (n)	24.4% (39)	41.7% (15)	24.4% (10)	36.1% (13)	29.7% (11)
Secondary drinking water source is improved, % (n)	8.8% (14)	27.8% (10)	9.8% (4)	22.2% (8)	16.2% (6)
Household obtains non-drinking water from improved source, % n)	65.6% (105)	66.7% (24)	63.4% (26)	66.7% (24)	59.5% (22)

[a] 4 households were dropped from analysis because of missing data on interruption duration.
[b] The truncated household water insecurity score excludes the interruption question.

Table S4. Prevalence and most probable number (MPN) of *E. coli* and cefotaxime-resistant *E. coli* by water source type

Water source type	N	Generic *E. coli*			Cefotaxime-resistant E.coli		
		N analyzed [a]	Prevalence % (n)	MPN/100 mL Mean (SD)	N analyzed [b]	Prevalence % (n)	MPN/100 mL Mean (SD)
**Piped sources**							
Piped into dwelling	20	20	25.0% (5)	135.6	19	5.3% (1)	0.53
Piped into yard/plot	45	44	43.2% (19)	14.0	42	0% (0)	0.50
Piped outside the compound	69	69	73.9% (51)	81.3	69	8.7% (6)	1.10
**Groundwater sources**							
Borehole	51	51	88.2% (45)	217.3	45	13.3% (6)	3.03
Protected spring	1	1	100% (1)	866.4	1	0% (0)	0.50
Protected dugwell	14	14	78.6% (11)	42.9	14	28.6% (4)	0.79
Unprotected dugwell	2	2	100% (2)	51.8	2	50.0% (1)	1.75
**Other sources**							
Surface water	2	2	100% (2)	17.6	2	0% (0)	0.50
Vendor water (kiosk)	30	30	53.3% (16)	43.5	29	3.4% (1)	0.81
Other	3	3	100% (3)	16.7	3	0% (0)	0.50

MPN: Most probable number, SD: Standard deviation

[a] 1 household missing water sample.

[b] 10 water samples not processed for cefotaxime-resistant *E. coli.*

Table S5. Study outcomes by binary interruption occurrence

			Interruption vs. No Interruption			
	No Interruption	Interruption	Unadjusted		Adjusted	
**Water quality (N=236)[a]**	**N=159**	**N=77**				
**Prevalence**	**% (n)**	**% (n)**	**PR (95% CI)**	**p-value**	**PR (95% CI)**	**p-value**
E. coli	66.0% (105)	64.0% (48)	0.98 (0.81, 1.20)	0.87	0.90 (0.74, 1.09)	0.28
Cefotaxime-resistant *E. coli* [b]	10.5% (16)	4.2% (3)	0.39 (0.21, 0.75)	0.08	0.47 (0.16, 1.33)	0.15
**Log10-MPN**	**Mean (SD)**	**Mean (SD)**	**ΔLog10 (95% CI)**	**p-value**	**ΔLog10 (95% CI)**	**p-value**
E. coli	0.8 (1.1)	0.7 (1.0)	-0.11 (-0.36, 0.14)	0.39	-0.17 (-0.41, 0.08)	0.19
Cefotaxime-resistant *E. coli* [b]	-0.2 (0.3)	-0.3 (0.2)	-0.04 (-0.13, 0.05)	0.34	-0.05 (-0.12, 0.02)	0.18
**Child health (N=292)[c]**	**N=202**	**N=90**				
**Prevalence**	**% (n)**	**% (n)**	**PR (95% CI)**	**p-value**	**PR (95% CI)**	**p-value**
Caregiver-defined diarrhea	16.3% (33)	31.1% (28)	**1.90 (1.07, 3.37)**	**0.03**	**1.85 (1.02, 3.37)**	**0.04**
WHO-defined diarrhea	13.9% (28)	22.2% (20)	1.60 (1.03, 2.83)	0.10	1.53 (0.72, 3.25)	0.27
ARI	40.6% (82)	57.8% (52)	**1.42 (1.09, 1.86)**	**0.01**	1.42 (0.97, 2.07)	0.07
ARI with fever	17.3% (35)	38.9% (35)	**2.24 (1.40, 3.59)**	**0.001**	**1.98 (1.09, 3.57)**	**0.02**
Rash	11.9% (24)	16.7% (15)	1.40 (0.75, 2.61)	0.29	1.32 (0.64, 2.73)	0.45
Child used antibiotics in last month	29.7% (60)	41.1% (36)	1.38 (0.99, 1.93)	0.06	1.19 (0.85, 1.66)	0.32
**Counts**	**Mean (SD)**	**Mean (SD)**	**CR (95% CI)**	**p-value**	**CR (95% CI)**	**p-value**
Number of times child used antibiotics	1.0 (2.6)	1.7 (3.7)	1.74 (0.99, 3.06)	0.06	1.39 (0.80, 1.99)	0.28
**Caregiver sress (N=237)**	**N=160**	**N=77**				
**Stress score**	**Mean (SD)**	**Mean (SD)**	**ΔPSS (95% CI)**	**p-value**	**ΔPSS (95% CI)**	**p-value**
PSS composite score [d]	18.8 (6.3)	21.6 (6.8)	**2.85 (1.36, 4.34)**	**<.0005**	1.15 (-0.44, 2.74)	0.16
**Prevalence**	**% (n)**	**% (n)**	**PR (95% CI)**	**p-value**	**PR (95% CI)**	**p-value**
High stress (PSS≥27) [d]	9.4% (15)	22.4% (17)	**2.39 (1.26, 4.53)**	**0.01**	1.69 (0.84, 3.37)	0.14
Often/always upset by unexpected events	13.1% (21)	23.4% (18)	**1.78 (1.07, 2.96)**	**0.03**	1.47 (0.83, 2.59)	0.19
Often/always unable to control things [d]	12.5% (20)	31.6% (24)	**2.53 (1.40, 4.56)**	**0.002**	**1.69 (1.01, 2.83)**	**0.05**
Often/always nervous or stressed	18.8% (30)	31.2% (24)	**1.66 (1.00, 2.77)**	**0.05**	1.32 (0.81, 2.16)	0.27
Never/rarely confident to solve problems	55.6% (89)	59.7% (46)	1.07 (0.86, 1.34)	0.52	1.11 (0.85, 1.44)	0.44
Never/rarely things go their way	54.4% (87)	57.1% (44)	1.05 (0.85, 1.30)	0.64	0.92 (0.75, 1.12)	0.41
Often/always unable to cope	15.6% (25)	26.0% (20)	**1.66 (1.01, 2.73)**	**0.05**	1.13 (0.67, 1.91)	0.64
Never/rarely able to control irritations	55.6% (89)	62.3% (48)	1.12 (0.90, 1.40)	0.32	1.18 (0.93, 1.51)	0.18
Never/rarely on top of things	53.1% (85)	55.8% (43)	1.05 (0.86, 1.28)	0.62	1.04 (0.89, 1.22)	0.60
Often/always angry	11.3% (18)	27.3% (21)	**2.42 (1.23, 4.79)**	**0.01**	1.75 (0.98, 3.14)	0.06
Often/always difficulties pile up	20.6% (33)	32.5% (25)	**1.57 (1.02, 2.42)**	**0.04**	1.18 (0.81, 1.73)	0.38

MPN: Most probable number, SD: Standard deviation, PR: Prevalence ratio, CR: Count ratio, PSS: Perceived stress scale, CI: Confidence interval.

[a] 1 household missing water sample.

[b] 10 water samples not processed for cefotaxime-resistant *E. coli.*

[c] N=292 children <5 years among 237 enrolled households.

[d] PSS composite score could not be calculated for 1 household that had a missing response to one question in the 10-question scale.

Table S6. Study outcomes by categorical interruption frequency. Rare refers to 1-2 interruptions, frequent refers to 3+ interruptions in the last month. Households with no interruptions are the reference group.

				Rare vs. No Interruption				Frequent vs. No Interruption			
	None	Rare	Frequent	Unadjusted		Adjusted		Unadjusted		Adjusted	
**Water quality (N=236)[a]**	**N=159**	**N=36**	**N=41**								
**Prevalence**	**% (n)**	**% (n)**	**% (n)**	**PR (95% CI)**	**p-value**	**PR (95% CI)**	**p-value**	**PR (95% CI)**	**p-value**	**PR (95% CI)**	**p-value**
E. coli	66.0% (105)	69.4% (25)	61.0% (25)	1.05 (0.86, 1.29)	0.63	0.89 (0.71, 1.12)	0.33	0.92 (0.70, 1.23)	0.53	0.90 (0.68, 1.19)	0.48
**Log10-MPN**	**Mean (SD)**	**Mean (SD)**	**Mean (SD)**	**ΔLog10 (95% CI)**	**p-value**	**ΔLog10 (95% CI)**	**p-value**	**ΔLog10 (95% CI)**	**p-value**	**ΔLog10 (95% CI)**	**p-value**
E. coli	0.8 (1.1)	0.8 (0.9)	0.6 (1.0)	0.00 (-0.23, 0.24)	0.97	-0.09 (-0.41, 0.24)	0.61	-0.21 (-0.55, 0.13)	0.22	-0.23 (-0.49, 0.03)	0.08
**Child health (N=292)[b]**	**N=202**	**N=39**	**N=51**								
**Prevalence**	**% (n)**	**% (n)**	**% (n)**	**PR (95% CI)**	**p-value**	**PR (95% CI)**	**p-value**	**PR (95% CI)**	**p-value**	**PR (95% CI)**	**p-value**
Caregiver-defined diarrhea	16.3% (33)	33.3% (13)	29.4% (15)	2.04 (0.95, 4.39)	0.07	**2.15 (1.05, 4.42)**	**0.04**	1.80 (0.93, 3.49)	0.08	1.57 (0.77, 3.23)	0.22
WHO-defined diarrhea	13.9% (28)	25.6% (10)	19.6% (10)	1.85 (0.89, 3.84)	0.10	1.95 (0.80, 4.73)	0.14	1.41 (0.72, 2.77)	0.31	1.15 (0.53, 2.50)	0.73
ARI	40.6% (82)	48.7% (19)	64.7% (33)	1.20 (0.81, 1.78)	0.36	1.11 (0.68, 1.79)	0.68	**1.59 (1.25, 2.03)**	**0.001**	**1.72 (1.18, 2.49)**	**0.005**
ARI with fever	17.3% (35)	33.3% (13)	41.1% (22)	1.92 (1.02, 3.64)	0.05	1.69 (0.86, 3.33)	0.13	**2.49 (1.62, 3.82)**	**0.001**	**2.20 (1.19, 4.06)**	**0.01**
Rash	11.9% (24)	23.1% (9)	11.8% (6)	**1.94 (1.17, 3.23)**	**0.01**	1.64 (0.72, 3.73)	0.24	0.99 (0.39, 2.53)	0.98	0.91 (0.32, 2.59)	0.86
Child used antibiotics in last month	29.7% (60)	41.0% (16)	41.2% (21)	1.38 (0.89, 2.14)	0.15	1.14 (0.71, 1.82)	0.59	1.39 (0.96, 2.00)	0.08	1.23 (0.88, 1.72)	0.23
**Counts**	**Mean (SD)**	**Mean (SD)**	**Mean (SD)**	**CR (95% CI)**	**p-value**	**CR (95% CI)**	**p-value**	**CR (95% CI)**	**p-value**	**CR (95% CI)**	**p-value**
Number of times child used antibiotics	1.0 (2.6)	1.6 (2.3)	1.8 (4.53)	1.62 (0.82, 3.19)	0.16	**1.98 (1.04, 3.78)**	**0.04**	1.83 (0.87, 3.84)	0.11	0.99 (0.43, 2.27)	0.97
**Caregiver sress (N=237)**	**N=160**	**N=36**	**N=41**								
**Stress score**	**Mean (SD)**	**Mean (SD)**	**Mean (SD)**	**ΔPSS (95% CI)**	**p-value**	**ΔPSS (95% CI)**	**p-value**	**ΔPSS (95% CI)**	**p-value**	**ΔPSS (95% CI)**	**p-value**
PSS composite score [c]	18.8 (6.3)	19.9 (6.28)	23.2 (6.73)	1.08 (-0.78, 2.94)	0.26	0.26 (-1.78, 2.30)	0.80	**4.44 (2.39, 6.50)**	**<.0005**	**2.06 (0.24, 3.89)**	**0.03**
**Prevalence**	**% (n)**	**% (n)**	**% (n)**	**PR (95% CI)**	**p-value**	**PR (95% CI)**	**p-value**	**PR (95% CI)**	**p-value**	**PR (95% CI)**	**p-value**
High stress (PSS≥27) [c]	9.4% (15)	11.1% (4)	32.5% (13)	1.19 (0.36, 3.85)	0.78	1.03 (0.33, 3.20)	0.96	**3.47 (1.77, 6.81)**	**<.0005**	**2.14 (1.01, 4.57)**	**0.05**
Often/always upset by unexpected events	13.1% (21)	16.7% (6)	29.3% (12)	1.27 (0.59, 2.71)	0.54	1.18 (0.62, 2.27)	0.61	**2.23 (1.20, 4.14)**	**0.01**	1.67 (0.88, 3.18)	0.12
Often/always unable to control things [c]	12.5% (20)	25.0% (9)	37.5% (15)	2.00 (0.93, 4.29)	0.08	1.45 (0.70, 2.99)	0.31	**3.00 (1.48, 6.07)**	**0.002**	1.95 (0.91, 4.17)	0.08
Often/always nervous or stressed	18.8% (30)	27.8% (10)	34.2% (14)	1.48 (0.79, 2.76)	0.22	1.30 (0.64, 2.66)	0.47	**1.82 (0.99, 3.34)**	**0.05**	1.34 (0.76, 2.34)	0.31
Never/rarely confident to solve problems	55.6% (89)	52.7% (19)	65.9% (27)	0.95 (0.65, 1.39)	0.79	1.08 (0.74, 1.57)	0.69	1.18 (0.92, 1.53)	0.20	1.14 (0.82, 1.58)	0.45
Never/rarely things go their way	54.4% (87)	58.3% (21)	56.1% (23)	1.07 (0.81, 1.43)	0.63	0.98 (0.71, 1.34)	0.89	1.03 (0.78, 1.36)	0.83	0.87 (0.67, 1.13)	0.28
Often/always unable to cope	15.6% (25)	19.4% (7)	31.7% (13)	1.24 (0.57, 2.71)	0.58	1.06 (0.45, 2.50)	0.90	**2.03 (1.13, 3.64)**	**0.02**	1.19 (0.64, 2.20)	0.58
Never/rarely able to control irritations	55.6% (89)	61.1% (22)	63.4% (26)	1.10 (0.79, 1.53)	0.57	1.15 (0.79, 1.67)	0.48	1.14 (0.88, 1.48)	0.33	1.22 (0.94, 1.59)	0.14
Never/rarely on top of things	53.1% (85)	55.6% (20)	56.1% (23)	1.06 (0.81, 1.36)	0.74	1.12 (0.87, 1.43)	0.38	1.06 (0.83, 1.35)	0.66	0.98 (0.76, 1.26)	0.88
Often/always angry	11.3% (18)	27.8% (10)	26.8% (11)	**2.47 (1.21, 5.04)**	**0.01**	**2.27 (1.15, 4.49)**	**0.02**	**2.38 (1.03, 5.54)**	**0.04**	1.40 (0.71, 2.77)	0.34
Often/always difficulties pile up	20.6% (33)	33.3% (12)	31.7% (13)	**1.62 (1.06, 2.45)**	**0.02**	1.30 (0.75, 2.23)	0.35	1.54 (0.86, 2.75)	0.15	1.10 (0.70, 1.71)	0.68

MPN: Most probable number, SD: Standard deviation, PR: Prevalence ratio, CR: Count ratio, PSS: Perceived stress scale, CI: Confidence interval.

[a] 1 household missing water sample.

[b] N=292 children <5 years among 237 enrolled households.

[c] PSS composite score could not be calculated for 1 household that had a missing response to one question in the 10-question scale.

Table S7. Study outcomes by categorical interruption duration. Short refers to below-median interruption duration (<1.3 days), long refers to above-median interruption duration (≥1.3 days). Households with no interruptions are the reference group.

				Short vs. No Interruption				Long vs. No Interruption			
	None	Short	Long	Unadjusted		Adjusted		Unadjusted		Adjusted	
**Water quality (N=232)[a,b]**	**N=159**	**N=36**	**N=37**								
**Prevalence**	**% (n)**	**% (n)**	**% (n)**	**PR (95% CI)**	**p-value**	**PR (95% CI)**	**p-value**	**PR (95% CI)**	**p-value**	**PR (95% CI)**	**p-value**
E. coli	66.0% (105)	66.7% (24)	62.2% (23)	1.01 (0.79, 1.30)	0.94	1.02 (0.80, 1.29)	0.88	0.94 (0.73, 1.21)	0.64	0.79 (0.60, 1.04)	0.09
**Log10-MPN**	**Mean (SD)**	**Mean (SD)**	**Mean (SD)**	**ΔLog10 (95% CI)**	**p-value**	**ΔLog10 (95% CI)**	**p-value**	**ΔLog10 (95% CI)**	**p-value**	**ΔLog10 (95% CI)**	**p-value**
E. coli	0.8 (1.1)	0.8 (1.0)	0.6 (0.9)	0.01 (-0.39, 0.40)	0.98	0.02 (-0.25, 0.29)	0.87	-0.23 (-0.48, 0.02)	0.08	-0.35 (-0.69, 0.01)	0.05
**Child health (N=287)[c]**	**N=202**	**N=45**	**N=40**								
**Prevalence**	**% (n)**	**% (n)**	**% (n)**	**PR (95% CI)**	**p-value**	**PR (95% CI)**	**p-value**	**PR (95% CI)**	**p-value**	**PR (95% CI)**	**p-value**
Caregiver-defined diarrhea	16.3% (33)	37.8% (17)	25.0% (10)	2.31 (1.10, 4.86)	**0.03**	2.09 (0.97, 4.49)	0.06	1.53 (0.84, 2.79)	0.17	1.52 (0.71, 3.26)	0.29
WHO-defined diarrhea	13.9% (28)	24.4% (11)	20.0% (8)	1.76 (0.91, 3.42)	0.09	1.58 (0.77, 3.26)	0.22	1.44 (0.69, 3.03)	0.32	1.41 (0.47, 4.20)	0.54
ARI	40.6% (82)	53.3% (24)	65.0% (26)	1.31 (0.95, 1.81)	0.10	1.29 (0.90, 1.84)	0.16	**1.60 (1.18, 2.17)**	**0.003**	1.60 (0.98, 2.61)	0.06
ARI with fever	17.3% (35)	33.3% (15)	47.5% (19)	**1.92 (1.14, 3.25)**	**0.02**	1.49 (0.78, 2.85)	0.23	**2.74 (1.74, 4.31)**	**<.0005**	**2.55 (1.47, 2.85)**	**<.0005**
Rash	11.9% (24)	20.0% (9)	12.5% (5)	1.68 (0.90, 3.15)	0.10	1.50 (0.62, 3.66)	0.37	1.05 (0.44, 2.51)	0.91	1.21 (0.44, 3.30)	0.71
Child used antibiotics in last month	29.7% (60)	42.2% (19)	42.5% (17)	1.42 (0.94, 2.14)	0.09	1.29 (0.90, 1.85)	0.17	1.43 (0.84, 2.44)	0.19	1.19 (0.66, 2.15)	0.56
**Counts**	**Mean (SD)**	**Mean (SD)**	**Mean (SD)**	**CR (95% CI)**	**p-value**	**CR (95% CI)**	**p-value**	**CR (95% CI)**	**p-value**	**CR (95% CI)**	**p-value**
Number of times child used antibiotics	1.0 (2.6)	1.2 (1.9)	2.4 (5.2)	1.24 (0.66, 2.34)	0.50	1.78 (0.92, 3.46)	0.09	**2.46 (1.17, 5.17)**	**0.02**	1.52 (0.51, 4.52)	0.45
**Caregiver stress (N=233)[a]**	**N=160**	**N=36**	**N=37**								
**Stress score**	**Mean (SD)**	**Mean (SD)**	**Mean (SD)**	**ΔPSS (95% CI)**	**p-value**	**ΔPSS (95% CI)**	**p-value**	**ΔPSS (95% CI)**	**p-value**	**ΔPSS (95% CI)**	**p-value**
PSS composite score [d]	18.8 (6.3)	22.2 (7.2)	21.4 (6.5)	**3.39 (1.09, 5.68)**	**0.004**	**1.80 (0.19, 3.42)**	**0.03**	**2.58 (0.58, 4.58)**	**0.01**	0.25 (-1.82, 2.32)	0.81
**Prevalence**	**% (n)**	**% (n)**	**% (n)**	**PR (95% CI)**	**p-value**	**PR (95% CI)**	**p-value**	**PR (95% CI)**	**p-value**	**PR (95% CI)**	**p-value**
High stress (PSS≥27) [d]	9.4% (15)	27.8% (10)	19.4% (7)	**2.96 (1.39, 6.30)**	**0.01**	1.90 (0.88, 4.12)	0.10	2.07 (0.96, 4.47)	0.06	1.54 (0.71, 3.35)	0.28
Often/always upset by unexpected events	13.1% (21)	25.0% (9)	21.6% (8)	**1.90 (1.07, 3.40)**	**0.03**	1.62 (0.89, 2.95)	0.11	1.65 (0.77, 3.54)	0.20	1.21 (0.57, 2.57)	0.62
Often/always unable to control things [d]	12.5% (20)	36.1% (13)	27.8% (10)	**2.89 (1.50, 5.55)**	**0.001**	**1.90 (1.08, 3.35)**	**0.03**	**2.22 (1.05, 4.68)**	**0.04**	1.45 (0.76, 2.76)	0.25
Often/always nervous or stressed	18.8% (30)	38.9% (14)	24.3% (9)	**2.07 (1.28, 3.35)**	**0.003**	**1.89 (1.03, 3.49)**	**0.04**	1.30 (0.58, 2.89)	0.52	0.81 (0.48, 1.38)	0.45
Never/rarely confident to solve problems	55.6% (89)	61.1% (22)	59.5% (22)	1.10 (0.79, 1.53)	0.58	1.09 (0.76, 1.56)	0.63	1.07 (0.81, 1.41)	0.64	1.04 (0.75, 1.43)	0.82
Never/rarely things go their way	54.4% (87)	58.3% (21)	54.1% (20)	1.07 (0.83, 1.39)	0.59	0.88 (0.68, 1.15)	0.36	0.99 (0.74, 1.33)	0.97	0.88 (0.68, 1.12)	0.29
Often/always unable to cope	15.6% (25)	27.8% (10)	21.6% (8)	1.78 (0.76, 4.16)	0.18	1.31 (0.62, 2.76)	0.48	1.38 (0.77, 2.50)	0.28	0.78 (0.35, 1.74)	0.55
Never/rarely able to control irritations	55.6% (89)	66.7% (24)	59.5% (22)	1.20 (0.97, 1.48)	0.10	1.16 (0.87, 1.55)	0.30	1.07 (0.81, 1.42)	0.64	1.18 (0.89, 1.56)	0.24
Never/rarely on top of things	53.1% (85)	55.6% (20)	56.8% (21)	1.05 (0.77, 1.41)	0.77	0.94 (0.67, 1.31)	0.72	1.07 (0.82, 1.39)	0.63	1.13 (0.93, 1.38)	0.22
Often/always angry	11.3% (18)	30.6% (11)	24.3% (9)	**2.72 (1.25, 5.92)**	**0.01**	**2.21 (1.12, 4.36)**	**0.02**	2.16 (0.98, 4.75)	0.06	1.29 (0.66, 2.54)	0.46
Often/always difficulties pile up	20.6% (33)	30.6% (11)	35.1% (13)	1.48 (0.83, 2.63)	0.18	1.12 (0.67, 1.87)	0.66	**1.70 (1.07, 2.72)**	**0.03**	1.25 (0.72, 2.17)	0.43

MPN: Most probable number, SD: Standard deviation, PR: Prevalence ratio, CR: Count ratio, PSS: Perceived stress scale, CI: Confidence interval.

[a] 4 households dropped from analysis because of missing data on intermittency duration.

[b] 1 household missing water sample.

[c] 5 children dropped from analysis because of missing data on intermittency duration.

[d] PSS composite score could not be calculated for 1 household that had a missing response to one question in the 10-question scale.

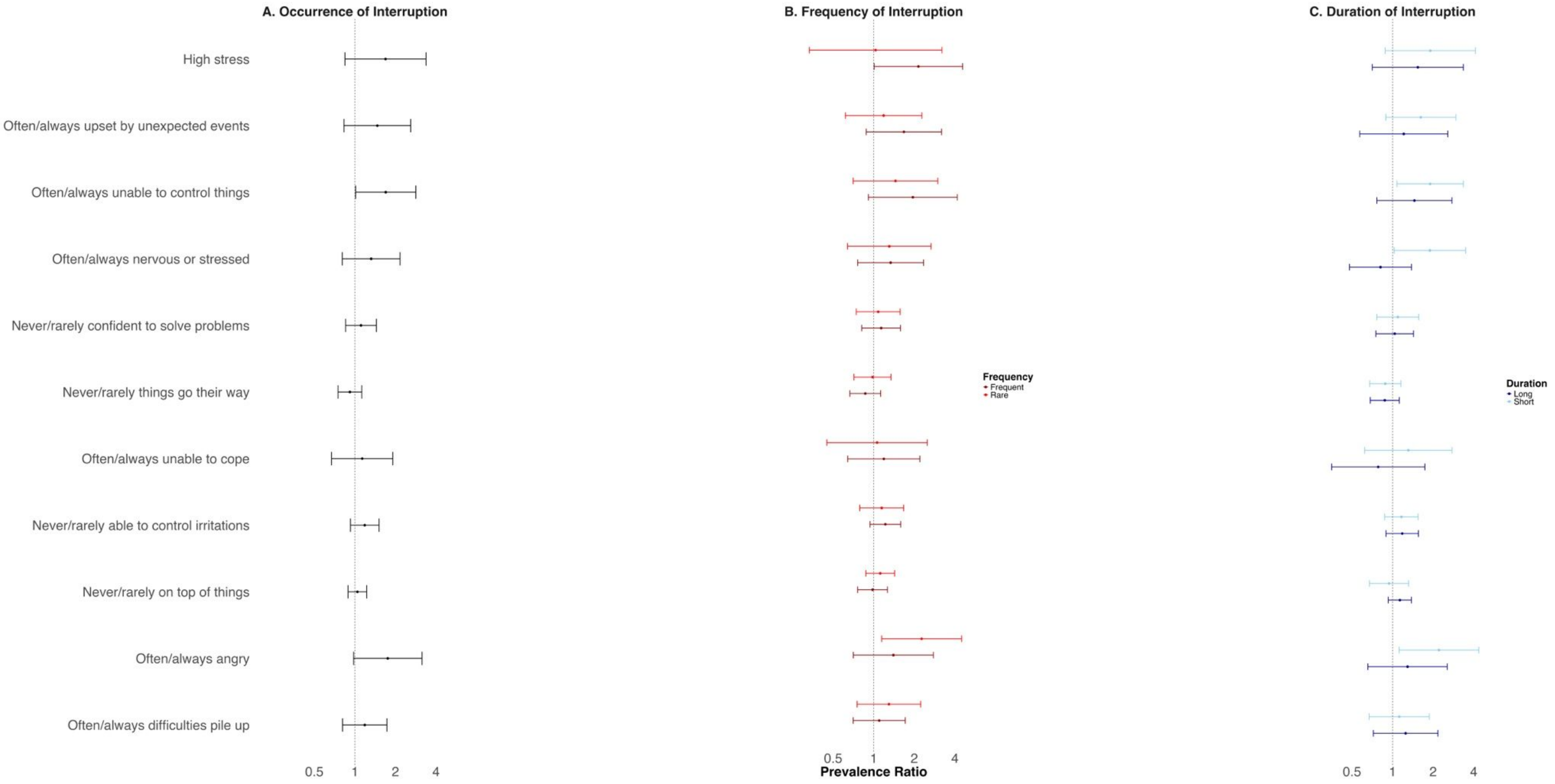

Fig S3. **Adjusted associations between caregiver stress indicators and (A) binary occurrence of water interruption in the last month, (B) categorical frequency of interruption in the last month (none, rare, frequent), and (C) categorical duration of the last experienced interruption (none, short, long).** Rare refers to 1-2 interruptions, frequent refers to 3+ interruption in the last month. Short refers to below-median interruption duration (<1.3 days), long refers to above-median interruption duration (≥1.3 days). Households with no interruptions are the reference group in all models. The circles denote point estimates for prevalence ratios, and the horizontal lines denote 95% confidence intervals. Models controlled for age of the respondent, age of the target child, total number of individuals living in household, respondent's highest level of education, highest level of education level for anyone in household, asset-based household wealth quintile, average weekly expenditures by household, household food insecurity score, whether household used piped water for their primary water source, whether household's designated handwashing station had soap, whether household had an improved latrine (as defined by the JMP), flooring material inside household, total number of animals in compound, and respondent's report of the last time it rained.
